# Short scales to assess cannabis-related problems: a review of psychometric properties

**DOI:** 10.1186/1747-597X-3-25

**Published:** 2008-12-02

**Authors:** Daniela Piontek, Ludwig Kraus, Danica Klempova

**Affiliations:** 1 IFT Institut für Therapieforschung, Parzivalstraße 25, D – 80804 München, Germany; 2 European Monitoring Centre for Drugs and Drug Addiction (EMCDDA), Rua da Cruz de Santa Apolonia 23–25, 1149-045 Lisbon, Portugal

## Abstract

**Aims:**

The purpose of this paper is to summarize the psychometric properties of four short screening scales to assess problematic forms of cannabis use: Severity of Dependence Scale (SDS), Cannabis Use Disorders Identification Test (CUDIT), Cannabis Abuse Screening Test (CAST) and Problematic Use of Marijuana (PUM).

**Methods:**

A systematic computer-based literature search was conducted within the databases of PubMed, PsychINFO and Addiction Abstracts. A total of 12 publications reporting measures of reliability or validity were identified: 8 concerning SDS, 2 concerning CUDIT and one concerning CAST and PUM. Studies spanned adult and adolescent samples from general and specific user populations in a number of countries worldwide.

**Results:**

All screening scales tended to have moderate to high internal consistency (Cronbach's α ranging from .72 to .92). Test-retest reliability and item total correlation have been reported for SDS with acceptable results. Results of validation studies varied depending on study population and standards used for validity assessment, but generally sensitivity, specificity and predictive power are satisfactory. Standard diagnostic cut-off points that can be generalized to different populations do not exist for any scale.

**Conclusion:**

Short screening scales to assess dependence and other problems related to the use of cannabis seem to be a time and cost saving opportunity to estimate overall prevalences of cannabis-related negative consequences and to identify at-risk persons prior to using more extensive diagnostic instruments. Nevertheless, further research is needed to assess the performance of the tests in different populations and in comparison to broader criteria of cannabis-related problems other than dependence.

## Introduction

In recent years, trend data from consecutive cross-sectional surveys in a variety of European countries have shown a general increase in the prevalence of cannabis use, particularly among the younger generations [1,2]. While, in most cases, consumption patterns remain experimental or occasional [3,4], the increasing prevalence may imply that a growing fraction of users will experience adverse consequences on mental and physical health or on a social level. Early identification of cannabis users at risk for negative consequences is of considerable value and the demand for appropriate and efficient screening instruments is increasing. A methodologically sound indicator would be useful for both epidemiological and clinical purposes. In the first case, screening scales could help to identify risk factors in order to prevent cannabis-related problems before they will cause serious health or other adverse consequences in a large number of people. This knowledge could guide the development and evaluation of public health policies. In the second case, based on the individual level of risk, users that have not yet entered the treatment system could be allocated to brief early interventions. Moreover, screening scales in clinical settings are a time and cost saving opportunity to identify at-risk persons prior to using more extensive diagnostic instruments.

Cannabis use has been associated with a range of adverse effects that can be categorized in acute effects (e.g. impaired attention, memory and psychomotor performance, road accidents), chronic health effects (e.g. bronchitis, depression, dependence syndrome) and social problems such as low educational attainment [5]. Furthermore, people with unstable health conditions have been found to take cannabis to reduce symptoms of depression, psychopathology or psychosocial distress [6,7]. But while there are clear definitions and diagnostic criteria for cannabis dependence and abuse, there is no agreed-upon concept for cannabis-related problems or so called "problem cannabis use" for individuals experiencing such acute, chronic or social adverse consequences without fulfilling the criteria for dependence. However, in recent times, different researchers work on the elaboration of a sub-threshold category "problem use" [8,9]. A number of other indicators have been discussed, among them intensity of use [10], consumption patterns (early morning smoking, intake to facilitate sleeping, using pipes or other facilities) or treatment demands [8], but to date none has reached common acceptance.

Despite these conceptual difficulties, there are different screening instruments used to measure negative consequences of cannabis use [11]. The Severity of Dependence Scale (SDS) [12] (see Appendix) is a 5-item scale that measures the degree of psychological dependence specifically related to the individuals' feeling of impaired control over and preoccupation and anxiety towards drug taking. The Cannabis Use Disorder Identification Test (CUDIT) [13] (see Appendix) screens for current cannabis use disorders (abuse or dependence) according to DSM-IV, whereas Problematic Use of Marijuana (PUM) [14] (see Appendix) measures ICD-10 harmful use along with problems in interpersonal relationships and psycho-physical functioning. Basically designed for adolescents or young adults, the Cannabis Abuse Screening Test (CAST) [15] (see Appendix) identifies patterns of cannabis use leading to negative consequences on a social or health level for the user himself or others. The major advantage of these four instruments is that they are brief and easy to administer. There are also other scales that measure cannabis-related problems, e. g. the Marijuana Screening Inventory (MSI) [16], the Substance Dependence Severity Scale (SDSS) [17], or the Cannabis Problems Questionnaire (CPQ) [18], but these are more extensive and too time-consuming for epidemiological purposes. Thus, MSI and CPQ consist of 31 and 29 items respectively taking about 10 minutes to complete and the SDSS additionally requires interviewer training and clinical experience.

In sum, there is considerable value in implementing screening instruments that are capable of detecting cannabis dependence or cannabis-related problems. Several short instruments have been developed and are available for routine use in general populations as well as in clinical settings. Despite of the high relevance, the number of research in this field is rather scarce. This review summarizes the psychometric properties of the four screening scales SDS, CUDIT, CAST and PUM by using peer-reviewed research to examine reliability and validity of these instruments in different samples. This overview may as well serve as a starting point for future research on the assessment of cannabis-related problems by identifying the major knowledge gaps.

## Methods

### Literature search

A systematic computer-based literature search was conducted within the databases of PubMed, PsycINFO and Addiction Abstracts. The four cannabis screening instruments ("Severity of Dependence Scale" in combination with "cannabis", as well as "Cannabis Abuse Screening Test", "Cannabis Use Disorders Identification Test" and "Problematic Use of Marijuana") were used as broad search terms in order to gather peer-reviewed articles citing any use of these measures with no specific time frame. This search was complemented by screening the World Wide Web as well as the literature lists of retrieved publications for further references on the subject. Articles were included if they evaluated at least one psychometric property concerning reliability or validity. Articles were excluded if the target questionnaires were used to only measure the validity of another instrument. Articles were also excluded if the instruments were used as screening tools to identify cannabis-related problems but no data on reliability or validity were presented.

The initial database search resulted in 64 publications referring to the four cannabis screening instruments in the title, the abstract or the keywords. These references were downloaded to a reference database and abstracts were screened to identify those relevant to the research question. Exclusion of non-relevant papers (i. e. predominantly biochemical or pharmacological topics, studies on the effects of cannabis use on different behavioural outcomes), non-empirical papers and studies only using but not validating the target instruments left eight publications for further consideration. Scanning the reference lists and the World Wide Web led to the identification of another four papers, adding up to a total of 12 selected publications for this review.

The majority of papers addressed the SDS [19-26]. Two studies evaluated the CUDIT [9,13]. CAST [15] and PUM [14] were dealt with once. Studies spanned adult and adolescent samples from seven different countries (Australia, Germany, France, Brazil, New Zealand, Switzerland and Poland). Samples from general and specific user populations were included. General characteristics of the selected publications are displayed in Table 1.

**Table 1:** Study characteristics

**Author (year)**	**Country**	**Scale**	**Assessment**	**Mode**	**Subjects**	**Sampling (response rate)**
**General population samples**						
Steiner et al. (2008)	Germany	SDS	Dependence	Questionnaire and telephone interview	456 adult cannabis users from population survey	Probabilistic population sample (45%, total sample)
Martin et al. (2006)	Australia	SDS	Dependence	Interview	100 adolescent cannabis users	Convenience sample
Kedzior et al. (2006)	Australia	SDS	Dependence	Interview	69 adult study participants (healthy and schizophrenia patients)	Convenience sample
Kedzior et al. (2007)	Australia	SDS	Dependence	Questionnaire	50 adult study participants (cannabis users)	Convenience sample
Okulicz-Kozaryn (2007)	Poland	PUM	Dependence	Questionnaire and interview	1277 adolescent cannabis users	Snowball sample
Legleye et al. (2007)	France	CAST	Social and health problems	Questionnaire	123 cannabis users from school survey	Non-probabilistic school sample (85%, total sample)
Annaheim et al. (2008)	Switzerland	CUDIT	Chronic, acute and social problems, Motivation	Telephone interview	593 adolescent cannabis users from population survey	Probabilistic population sample (61.6%, total sample)

**Specific user samples**						
Swift, Hall et al. (1998)	Australia	SDS	Dependence	Interview	143 long-term cannabis users	Snowball sample
Swift, Copeland & Hall (1998)	Australia	SDS	Dependence	Questionnaire and interview	200 long-term cannabis users	Convenience sample
Hides et al. (2007)	Australia	SDS	Dependence	Interview	153 in-patients with schizophrenia	Convenience sample
Ferri et al. (2000)	Brazil	SDS	Dependence	Interviewer-administered questionnaire	347 regular users of cocaine	Convenience sample
Adamson & Sellman (2003)	New Zealand	CUDIT	Dependence	Questionnaire and interview	53 out-patients with alcohol dependence (cannabis users)	Convenience sample

### Data extraction

Data extraction for this review followed predefined criteria considered important in the evaluation of screening instruments and was guided by a standardised documentation sheet. As indicators of reliability, internal consistency and test-retest reliability were considered. Construct validity was evaluated by considering the scales' factor structure as well as correlations with related constructs (convergent validity). Concerning criterion validity, sensitivity, specificity, positive predictive value (PPV) and negative predictive value (NPV) were included. Moreover, results of Receiver Operating Characteristics (ROC) computing cut-off values are reported.

For the evaluation of psychometric properties of screening instruments it is important to differentiate between studies using samples from general populations and those using specific user populations, as this directly affects several psychometric indices. For example, the prevalence of a disorder within the study population will influence the ability of any screening test to detect it accurately. Thus, if cannabis dependence is rare (in general population samples), a negative test will more likely indicate no disease (high NPV) and a positive test will less likely indicate the presence of the disease (low PPV) [27] than when it is common, e.g. in samples of heavy cannabis users.

## Results

### Reliability

#### Internal consistency

##### General population

Five studies evaluated the internal consistency of the screening scales in general population samples using Cronbach's coefficient α (Table 2). Analyses suggest high consistency of at least .72 for all four instruments, with the highest value being for PUM [14] and the lowest value for CUDIT [9]. On the other hand, item-total correlations reported in two studies were only moderate varying remarkably across items. Thus, kappa indices were low to moderate for single pairs of items of the CAST [15]. In addition, corrected item-total correlations of the CUDIT were modest for most items, while inacceptable for items 2 (usual hours being stoned) and 9 (injuries) [9].

**Table 2:** Reliability of cannabis screening instruments

**Scale**	**Study**	**Internal consistency**	**Test-retest reliability**	**Item total correlation**
**General population samples**				
SDS	Steiner et al. (2008)	.80		
SDS	Martin et al. (2006)	.83	.88 (total), .69 to .85 (items)	
CAST	Legleye et al. (2007)	.81 [.74 for cannabis users]		.26 to .56 (pairs of items)
CUDIT	Annaheim et al. (2008)	.72		.07 to .55
PUM	Okulicz-Kozaryn (2007)	.92		

**Specific user samples**				
SDS	Swift, Hall et al. (1998)	.72		
SDS	Hides et al. (2007)	.81		
SDS	Ferri et al. (2000)	.78	.74 (total), .46 to .76 (items)	
CUDIT	Adamson & Sellman (2003)	.78		.44 to .77

##### Specific user samples

Data from studies on specific cannabis users support high internal consistency for the SDS (Table 2). This holds true for samples of long-term cannabis users (at least 10 years with weekly use) [26], schizophrenia patients (cannabis use in the last 12 months) [20] and regular users of cocaine [19]. Additionally, a study of out-patients with alcohol dependence found good internal consistency for the CUDIT [13]. Item-total correlations for nine out of ten individual items were moderate to high, but could not be calculated for item 9 (injuries) as there were no positive responses to this question.

#### Test-retest reliability

##### General population

Only one paper concerning the SDS included temporal reliability coefficients (Table 2) in an adolescents sample from the general population [23]. Intra-class correlation was satisfactory for both the scale's total score and the single items over a one week period.

##### Specific user samples

Three day test-retest correlations of SDS were reported for a sample of cocaine dependent patients [19]. Reliability was good for the total score and moderate to good for the single items of the scale.

### Validity

#### Construct validity

##### General population

Factor analyses of the SDS, CAST and CUDIT have been reported in four studies using adolescent and adult general population samples [13,15,23,24]. In all cases analyses resulted in a single factor solution that accounted for a substantial portion of the total variance (53% and 57.6%). Individual factor loadings were moderate to high (.41 to .87) for most items (except for CUDIT item 9 performing very weakly).

Construct validity can also be assessed by measuring the extent to which a scale correlates positively with other measures that address the same construct (convergent validity). Thus, moderate to high positive correlations (r = .32 to .76) were found between the SDS total score and frequency of cannabis use [23,24], amount of cannabis use [24] and the number of DSM-IV dependence criteria [23,24]. In addition, Legleye et al. [15] reported several correlations of the CAST score with psycho-pathological dimensions of the Problem Oriented Screening Instrument for Teenagers (POSIT) [28] that measures psychological, physical and social health impairments. Students with higher CAST scores reported worse physical and mental health and more school problems.

##### Specific user samples

Factor structure of the SDS has also been examined in samples which included participants with drug dependence, psychosis and long-term cannabis dependence [19,20,26]. Each showed that a single factor accounted for a considerable portion of the total variance (48.4 – 56.8%). Consistently, all five items of the SDS had high positive correlations on the factor score of greater than .50. Convergent validity of the SDS was demonstrated by reporting moderate to high significant correlations between the scale's total score and the estimated quantity of cannabis use, frequency of use and the number of DSM-IV dependence criteria [19,21]. In the study of Swift, Hall et al. [26] significant correlations between SDS and quantity and frequency of use as well as age at first use and total duration of use were not found. However, in their sample of long-term cannabis users they could show a substantial agreement between the SDS score and the respondents' belief that their cannabis use caused problems (kappa = .44).

#### Criterion validity

The selection of a specific external criterion for validation is an important methodological consideration that influences the interpretation of the results. Since a common definition of cannabis-related problems does not exist, the current "gold standard" for assessing criterion validity is to evaluate the screening instruments against DSM-IV diagnosis of cannabis dependence [29]. Alternatively, it is possible to cross-validate screening instruments against broader concepts of problem use, like acute consequences or psychosocial problems related to cannabis consumption.

##### General population

Most studies reporting measures of criterion validity used a dependence diagnosis (DSM-IV or ICD-10) retrieved from a clinical interview as an external criterion. Altogether, SDS and PUM were found to have a good ability to discriminate between dependent and non-dependent individuals, correctly classifying at least 85% of cases (Table 3). Though different cut-off points have been reported depending on the study sample, all evaluations revealed high values of specificity and usually lower, but still high, levels of sensitivity. As an exception, the study by Steiner et al. showed much higher sensitivity than specificity [24]. Moreover, the positive predictive power of the SDS is very low.

**Table 3:** Criterion validity of cannabis screening instruments

**Scale**	**Study**	**Criterion**	**Cut-off**	**Sensitivity**	**Specificity**	**Positive predictive value**	**Negative predictive value**	**Area under the curve**
**General population samples**								
SDS	Steiner et al. (2008)	DSM-IV dependence	2 [men: 4]	93.6% [81.0%]	74.0% [86.9%]	28.8%	99.0%	.92
SDS	Martin et al. (2006)	DSM-IV dependence	4	65.1%	94.3%			.85
SDS	Kedzior et al. (2007)	Any DSM-IV or ICD-10 diagnosis		72.7%^a^	96.4%^a^	94.1%^a^	81.8%^a^	
PUM	Okulicz-Kozaryn (2007)	ICD-10 dependence	2	80.9%	87.5%	86.6%	82.2%	.91
CAST	Legleye et al. (2007)	POSIT^c^	4	92.9%	81.4%	45.8%^b^	96.5%^b^	.92
CUDIT	Annaheim et al. (2008)	Use at school	7	71.1%	64.2%	14.1%^b^	96.4%^b^	.74
		Use and drive	5	66.7%	58.9%	29.6%^b^	83.3%^b^	.63
		Depression	5	57.8%	50.4%	43.8%^b^	64.2%^b^	.57
		Use to cope	5	75.0%	58.9%	49.2%^b^	81.6%^b^	.76
		Self-evaluation	7	71.3%	69.7%	36.6%^b^	91.0%^b^	.77

**Specific user samples**								
SDS	Swift, Copeland & Hall (1998)	DSM-III-R dependence	3	64.0%	82.0%			.77
SDS	Hides et al. (2007)	DSM-IV dependence	2	86.0%	83.0%	83.0%	86.0%	
CUDIT	Adamson & Sellman (2003)	DSM-IV dependence	8	73.3%	94.7%	84.6%	90.0%	.87

^a ^Computed by the authors. ^b ^Received on request

^c ^Problem Oriented Screening Instrument for Teenagers [28] measures psychological, physical and social problems.

Two studies have examined the scales for possible sex and age differences. PUM seems equally appropriate for males and females as well as for different age groups [14]. The original cut-off score could also be maintained in a subsample for which cannabis was the first choice drug. Validity of the SDS did also not differ between several ages [24]. However, choosing a higher cut-off increased sensitivity and specificity for males as compared to females.

In contrast to previous studies, two projects did not use DSM-IV or ICD-10 diagnoses as the "gold standard" to compare with but some broader concepts of problem use. Thus, the drug abuse section of the POSIT questionnaire was used to cross-validate CAST scores [15] and several single-item indicators of cannabis-related problems were used to validate CUDIT [9]. In this context, CAST proved to be effective when screening for high risks of abuse showing high values of sensitivity, specificity and predictive power (Table 3). CUDIT revealed quite favourable levels of sensitivity and lower, but in most cases still acceptable, specificity. As in the Steiner et al. study [24], positive predictive value of the scale was low due to the low base rate of cannabis use in the overall sample.

##### Specific user samples

Three papers reported the validity of the SDS und CUDIT against DSM diagnoses in cannabis users (Table 3). Again, different cut-off points were found depending on the study sample. Sensitivity, specificity and predictive power of the SDS are comparable to those reported in general population samples [20,25]. CUDIT performed better in an alcohol-dependent sample [13] as compared to the general population study mentioned above. There are no studies evaluating possible subgroup differences (sex or age) or applying other external criteria than dependence diagnoses.

## Discussion

Aim of this paper was to summarize the psychometric properties of four short screening scales to assess cannabis-related disorders or problems (SDS, CUDIT, CAST, PUM). A total of 12 studies from a variety of countries using diverse samples from general and specific user populations have been identified. While the SDS has been well studied in different samples, research on the other three instruments is limited. Psychometric properties of all scales varied depending on the study population and standards used for validity assessment, and therefore, comparisons between scales and studies are limited.

The results of our review suggest that the SDS is a valid diagnostic instrument for both general and specific user populations. Its diagnostic potential as a screen for identifying individuals with symptoms of substance use dependence has been shown not only for cannabis but also for cocaine [30], amphetamines [31], benzodiazepines [32], and most recently alcohol [33]. However, a standard diagnostic cut-off does not exist. The cut-off points reported vary between 2 and 4 across different studies. Specific analyses for sub-populations (different age groups, sex) also revealed differences. This implies that cut-off scores need to be defined independently for different populations.

The available evidence on psychometric properties comes primarily from studies evaluating SDS against cannabis dependence diagnoses. Only two studies used other criteria, like psychosocial consequences or a self-evaluation of problem cannabis use [9,15]. Both approaches have their individual benefits. The major advantage in using the "gold standard" cannabis dependence is to rely on an internationally acknowledged construct with predefined criteria and operationalizations. On the other hand, referring to criteria other than dependence symptoms allows a broader view on cannabis-related problems that might be helpful in detecting people who experience acute or chronic negative consequences without fulfilling the criteria for dependence.

The instruments most recently evaluated [9,15] assess cannabis-related problems namely social or health problems (CAST) and in addition motivational aspects of cannabis use (CUDIT). The psychometric properties of the CAST are comparable to the SDS, while the performance of the CUDIT is moderate. In particular, two items – *cannabis related injury *(item 9) and *usual hours being stoned *(item 2) – performed very weakly in tests of reliability and construct validity. These questions need to be revised or deleted from the scale especially as there are hardly positive responses in the samples studied.

There are a number of methodological considerations concerning the evaluation of screening scales that, independent of the assessment criteria, need to be further elucidated. First, the choice of an optimal cut-off largely depends on the context of the study and the specific task at hand [34]. For example, using the SDS as a screening tool for cannabis dependence in a clinical setting, it may be preferable to maximize the ability to detect people who are at risk for dependence (i. e. use a lower cut-off) at the expense of decreased specificity. On the other hand, it may be more important to minimize costs, i.e. to avoid selecting people for treatment who do not need it. In this case, it may be more appropriate to use a higher cut-off with increased specificity and decreased sensitivity. In research settings, where the main interest is to estimate prevalence, the optimal approach would be to take a value that offers the best balance between sensitivity and specificity.

Second, a clear understanding of the way in which diagnostic accuracy may be affected by the population to which a screening test is applied is required. As noted earlier, the prevalence of a cannabis-related disorder or problem will affect the ability of any screening test to detect it accurately. Predictive power will be low when the prevalence is low, even if sensitivity and specificity of the instrument are high [27,35]. On population level, this effect may lead to a non-negligible overestimation of the prevalence. For example, positive predictive values of SDS and CUDIT tested in truly probabilistic samples [9,24] were rather low with 14.1% to 49.2%. Other studies used targeted samples of preselected cannabis users (e.g. recruited through magazines, or snowball sampling) [14,22,23] which resulted in higher positive predictive values up to 94.1%.

Third, the eligibility of dependence diagnoses as the "gold standard" itself needs to be discussed. There is still a controversy about the way in which the drug (and especially the cannabis) dependence syndrome should be operationalized in future diagnostic systems like DSM-V, which is currently being prepared. For example, it has been argued that the rule of three criteria that has been defined for alcohol and opiate dependence may be over-inclusive for cannabis [36,37]. Others emphasize psychological components or behavioural indicators of dependence [37] or propose dimensional equivalents for the so far categorically defined substance use disorders [38].

There has been some discussion on the applicability of screening instruments in different settings (research vs. clinical). For example, in the original publication Gossop et al. [12] explicitly recommend the SDS for research purposes and not for clinical use. In recent times however, increasing evidence suggests its utility as a clinical *and *research tool [33]. Screens can be specifically useful in clinical settings as their results can determine further steps in the individual assessment or treatment planning. Those patients with a positive screening result can be referred to more extensive diagnostic assessments of cannabis dependence (for example CIDI). Patients, for whom the CIDI assessment does not confirm the screening result, may still experience negative consequences from their cannabis use. These individuals do not need full treatment but may be referred to brief interventions. In the case of cannabis, further studies need to assess the performance of screening scales in different clinical settings.

In general, users of screening tests should be cautious in addressing the identified individuals as problem cannabis users without considering the particular problems the test is referring to. On the other hand, depending on the purpose a screen is used for (e.g., prevalence estimation, clinical assessment) the recipients of the results should be made aware of the psychometric properties of the test. The choice of the cut-off determines the proportion of targeted but not identified individuals as well as the proportion of individuals who although identified do not fulfil the reference criteria.

Further research and developments regarding screening instruments are required in order to improve the assessment of cannabis-related problems. As mentioned earlier, at least CUDIT may need some revision because of the very weak performance of several items in psychometric analyses. Additional research should evaluate the usefulness of screening scales in different settings and for sub-populations of particular interest. With regard to adolescents, current projects in France and Spain investigate the performance of CAST in different samples. In Australia, attempts are made to develop a new screen intended to identify current and potential problems related to cannabis use on population level.

## Conclusion

Identifying people who show symptoms of dependence or experience other acute or chronic problems due to cannabis use is important both in the general population and the clinical setting. In general, all four screening scales included in our review yielded satisfactory measures of reliability and validity. Furthermore, these tests are brief and easy to administer and have been used in a variety of populations. The SDS is a valid and viable instrument for screening cannabis dependence. It may be used as an adjunct to more extensive diagnostic interments. Some progress has been made in the development of short instruments to assess negative consequences of cannabis use other than dependence. Research questions remain with regard to the performance of the instruments in representative population samples and in comparison to broader criteria of cannabis-related problems.

## Competing interests

The authors declare that they have no competing interests.

## Authors' contributions

DP collected and analyzed the data and drafted the manuscript. LK was involved in interpreting the results and drafting the manuscript. DK participated in the interpretation of the results and the critical revision of the manuscript. All authors read and approved the final manuscript.

## Appendix

### Severity of Dependence Scale (SDS)

During the past year...

(1) Did you think your use of cannabis was out of control?

never/almost never (0) – sometimes (1) – often (2) – always/nearly always (3)

(2) Did the prospect of missing a dose of cannabis makes you anxious or worried?

never/almost never (0) – sometimes (1) – often (2) – always/nearly always (3)

(3) Did you worry about your use of cannabis?

never/almost never (0) – sometimes (1) – often (2) – always/nearly always (3)

(4) Did you wish you could stop the use of cannabis?

never/almost never (0) – sometimes (1) – often (2) – always/nearly always (3)

(5) How difficult did you find it to stop, or go without cannabis?

Not difficult (0) – quite difficult (1) – very difficult (2) – impossible (3)

From: Gossop M, Darke S, Griffith P, Hando J, Powis B, Hall W, Strang J in *Addiction *1995, 90(5), 607 – 614.

### Cannabis Use Disorders Identification Test (CUDIT)

Over the past 6 months...

(1) How often did you use cannabis?

Never (0) – monthly or less (1) – 2 – 4 times a month (2) – 2 – 3 times a week (3) – 4 or more times a week (4)

(2) How many hours were you "stoned" on a typical day when you had been using cannabis?

1 or 2 (0) – 3 or 4 (1) – 5 or 6 (2) – 7 to 9 (3) – 10 or more (4)

(3) How often were you "stoned" for 6 or more hours?

Never (0) – less than monthly (1) – monthly (2) – weekly (3) – daily or almost daily (4)

(4) How often did you find that you were not able to stop using cannabis once you had started?

Never (0) – less than monthly (1) – monthly (2) – weekly (3) – daily or almost daily (4)

(5) How often did you fail to do what was normally expected from you because of using cannabis?

Never (0) – less than monthly (1) – monthly (2) – weekly (3) – daily or almost daily (4)

(6) How often did you needed to use cannabis in the morning to get yourself going after a heavy session of using cannabis?

Never (0) – less than monthly (1) – monthly (2) – weekly (3) – daily or almost daily (4)

(7) How often did you have a feeling of guilt or remorse after using cannabis?

Never (0) – less than monthly (1) – monthly (2) – weekly (3) – daily or almost daily (4)

(8) How often have you had a problem with your memory or concentration after using cannabis?

Never (0) – less than monthly (1) – monthly (2) – weekly (3) – daily or almost daily (4)

(9) Have you or someone else been injured as a result of your use of cannabis?

No (0) – yes (4)

(10) Has a relative, friend or doctor or other health worker been concerned about your use of cannabis or suggested you cut down?

No (0) – yes (4)

From: Adamson SJ, Sellman JD in *Drug Alcohol Rev *2003, 22, 309 – 315.

### Cannabis Abuse Screening Test (CAST)

(1) Have you ever smoked cannabis before midday?

never (0) – rarely (0) – from time to time (0) – fairly often (1) – very often (1)

(2) Have you ever smoked cannabis when you were alone?

never (0) – rarely (0) – from time to time (0) – fairly often (1) – very often (1)

(3) Have you ever had memory problems when you smoked cannabis?

never (0) – rarely (0) – from time to time (0) – fairly often (1) – very often (1)

(4) Have friends or members of your family ever told you that you ought to reduce your cannabis use?

never (0) – rarely (0) – from time to time (0) – fairly often (1) – very often (1)

(5) Have you ever tried to reduce or stop your cannabis use without succeeding?

never (0) – rarely (0) – from time to time (0) – fairly often (1) – very often (1)

(6) Have you ever had problems because of your use of cannabis (argument, fight, accident, bad result at school, etc.)?

never (0) – rarely (0) – from time to time (0) – fairly often (1) – very often (1)

From: Legleye S, Karila L, Beck F, Reynaud M in *J Subst Use *2007, 12(4), 233 – 242.

### Problematic Use of Marijuana (PUM)

(1) Have you ever skipped school classes or came late to school because of cannabis use?

Yes – no

(2) Have you had a serious argument with family members because of your cannabis use?

Yes – no

(3) Have you had a serious argument with friends because of your cannabis use?

Yes – no

(4) Have you ever bought cannabis yourself?

Yes – no

(5) Do you have more and more problems In studying and understanding new information?

Yes – no

(6) Have you ever used cannabis when you were alone?

Yes – no

(7) Do you often feel desire for cannabis?

Yes – no

(8) Have you ever spent so much money on cannabis that you had to resign from other things or activities?

Yes – no

From: Okulicz-Kozaryn K in *Postepy Psychiatrii i Neurologii *2007, 16(2),105 – 111. Translation by the original author.
